# RETRACTION: Risk Factors Related to Surgical Wound Infection After Caesarean Section: A Systematic Review and Meta‐Analysis

**DOI:** 10.1111/iwj.70432

**Published:** 2025-03-26

## Abstract

**RETRACTION:** ZhuD. , WuY. , FanL. , and LiuX. , “Risk Factors Related to Surgical Wound Infection After Caesarean Section: A Systematic Review and Meta‐Analysis,” International Wound Journal 21, no. 2 (2024): e14580, 10.1111/iwj.14580.PMC1083038842052980

The above article, published online on 31 January 2024, in Wiley Online Library (http://onlinelibrary.wiley.com/), has been retracted by agreement between the journal Editor in Chief, Professor Keith Harding; and John Wiley & Sons Ltd. Following an investigation by the publisher, all parties have concluded that this article was accepted solely on the basis of a compromised peer review process. In addition, the investigation found there was significant unattributed textual overlap between this article and a previously published article (Yang et al. 2023 [https://doi.org/10.1111/iwj.14420]). The editors have therefore decided to retract the article. The authors disagree with the retraction.

**RETRACTION:** D. Zhu , Y. Wu , L. Fan , and X. Liu , “Risk Factors Related to Surgical Wound Infection After Caesarean Section: A Systematic Review and Meta‐Analysis,” International Wound Journal 21, no. 2 (2024): e14580, 10.1111/iwj.14580.PMC1083038842052980

The above article, published online on 31 January 2024, in Wiley Online Library (http://onlinelibrary.wiley.com/), has been retracted by agreement between the journal Editor in Chief, Professor Keith Harding; and John Wiley & Sons Ltd. Following an investigation by the publisher, all parties have concluded that this article was accepted solely on the basis of a compromised peer review process. In addition, the investigation found there was significant unattributed textual overlap between this article and a previously published article (Yang et al. 2023 [https://doi.org/10.1111/iwj.14420]). The editors have therefore decided to retract the article. The authors disagree with the retraction.

